# Association between retinal sensitivity and the presence of quiescent choroidal neovascularization in pachychoroid diseases

**DOI:** 10.1371/journal.pone.0271543

**Published:** 2022-07-26

**Authors:** Rion Ozawa, Keiko Azuma, Yoko Nomura, Hiroshi Murata, Ryo Asaoka, Kohdai Kitamoto, Kohei Ueda, Tatsuya Inoue, Ryo Obata

**Affiliations:** 1 Graduate School of Medicine and Faculty of Medicine, Department of Ophthalmology, The University of Tokyo, Tokyo, Japan; 2 Department of Ophthalmology, Seirei Hamamatsu General Hospital, Seirei Christopher University, Shizuoka, Japan; 3 Department of Ophthalmology and Micro-Technology, Yokohama City University School of Medicine, Minami-ku, Yokohama, Kanagawa, Japan; Biruni University Medicana International Hospistal, TURKEY

## Abstract

This study was conducted to examine retinal sensitivity (RS) in eyes with pachychoroid diseases and to analyze its association with the presence or absence of quiescent choroidal neovascularization (CNV), that can be protective against retinal dysfunction or atrophy in other macular diseases such as age-related macular degeneration. A total of 12 eyes of 12 patients aged ≥45 years having the characteristic findings of central serous chorioretinopathy but not presenting any exudative changes were included in this study. Choroidal vascular hyper permeability (CVH) was identified by indocyanine green angiography, and the presence or absence of CNV was evaluated by optical coherence tomography angiography. RS at 68 points was examined by microperimetry. The average RS corresponding to within and outside CVH was compared. The association between the difference in RS and the presence or absence of CNV was also analyzed. CNV was detected in six eyes (50%). In eyes without CNV, the RS within CVH was similar compared with that outside CVH. However, in eyes with CNV, the RS within CVH was significantly decreased compared with that outside CVH. Multiple regression analysis revealed the presence of CNV as an independent factor associated with RS. In eyes with pachychoroid diseases, RS decreased within the CVH area under the coexistence of nonexudative CNV.

## Introduction

Pachychoroid diseases [1] are a series of clinical entities that share some characteristic findings in the retina or the choroid [2,3]. The most important finding is a focally or diffusely thickened choroid that contains dilated choroidal vessels and attenuated choriocapillaris accompanied with abnormalities of the retinal pigment epithelium (RPE) [1]. The lesion is often characterized by choroidal vascular hyper permeability (CVH) revealed by indocyanine green angiography (ICGA). The clinical entities include pachychoroid pigment epitheliopathy (PPE) [4,5], chronic central serous chorioretinopathy (CSC), pachychoroid neovasculopathy [6,7], polypoidal choroidal vasculopathy (PCV) [6,7], and pachychoroid geographic atrophy [8,9]. During the course of these diseases [10,11], they present exudative changes such as serous detachment of the retina or the RPE with or without choroidal neovascularization (CNV) and atrophic changes at the choriocapillaris, the RPE, or the sensory retina. The area of CVH has been reported to be anatomically linked with these changes [12,13]. To date, increasing evidence has suggested that pachychoroid diseases should be distinguished from age-related macular degeneration (AMD), which exhibits similar exudative or atrophic changes in the posterior retina [14].

Recent advancement of imaging technology, especially that of optical coherence tomography angiography (OCTA), has contributed to increased detection of CNV or macular neovascularization (MNV), especially quiescent or nonexudative CNV or nonexudative MNV, i.e., neovascular tissue not accompanied with exudative changes. In a previous review, nonexudative MNV was detected in 6%–27% of the fellow eyes with AMD [15]. Nonexudative MNV could be a precursor for exudative AMD, but it may retard the growth of geographic atrophy and could have a protective effect on retinal function, probably at least in the stage where the lesion was quiescent [15,16].

Meanwhile, several studies have reported the presence of nonexudative CNV in pachychoroid diseases [17,18]. The presence of nonexudative CNV could also be a risk factor for the development of exudative changes in CSC [19] and PCV [20]. However, whether nonexudative CNV affects the retinal function is not clearly understood.

A previous study [21] showed that eyes with chronic CSC complicated with CNV demonstrated worse visual or reading acuity than those without CNV. However, considering that the lesion does not necessarily involve the fovea, it is important to not only compare visual acuity but also analyze retinal sensitivity (RS) at the lesion area. Nevertheless, to the best of our knowledge, there has been no published report investigating the association between RS and the presence or absence of quiescent CNV.

Therefore, in the present study, we investigated RS by microperimetry in eyes with pachychoroid diseases. We then evaluated the local function corresponding to within or outside the lesion area. In addition, we examined the association between the presence or absence of CNV and the changes in RS.

## Methods

This retrospective study was conducted according to the tenets of the Declaration of Helsinki and was approved by the institutional review board (IRB) of the University of Tokyo. Written informed consent was not required by the IRB, but participants who did not grant authorization to use their medical records for the research were excluded from the study.

### Participants

We retrospectively reviewed the medical charts of consecutive patients, including 12 eyes of 12 patients with chronic CSC who visited the University of Tokyo Hospital from April 2018 to July 2019. All subjects met the following criteria: (1) patients were diagnosed with CSC with CVH lesions in the examined eye, and (2) patients had not shown any exudative changes or hemorrhage for more than 6 months by the time of RS examination. Patients who were treated with photodynamic therapy or antivascular endothelial growth factor therapy were not included. Patients complicated with other retinal diseases such as diabetic retinopathy, retinal vascular diseases, myopic maculopathy, glaucoma, and significant cataract that could affect visual function were also excluded.

### Multimodal imaging

All patients were examined by fluorescein angiography and ICGA (Heidelberg Retina Angiograph 2; Heidelberg Engineering, Heidelberg, Germany), spectral-domain optical coherence tomography (OCT) (Heidelberg Engineering GmbH, Dossenheim, Germany), and OCTA for diagnosis. Central choroidal thickness (CCT) was also measured on OCT based on enhanced depth images. By reviewing the OCT images obtained from the initial visit to six months before the examination, the maximum height and the duration of SRD was assessed. Additionally, using OCT we assessed the integrity of ellipsoid zone (EZ) and interdigitation zone (IZ) above the CVH area. The disruption of EZ or IZ were suggested to be impairment of the photoreceptor. Previously, Hood et al. reported that the point where EZ/IZ disappears provides a structural marker for visual field (VF) [22].

### OCTA measurement

OCTA images were acquired using the Angiovue software of RTVue XR Avanti (Optovue, Inc., Fremont, CA). OCTA was performed using the split-spectrum amplitude-decorrelation angiography system. OCTA images were obtained with a 3 × 3 mm and 6 × 6 mm macular cube centered on the lesion, and an automated segmentation layer was set to both the outer retina and the choriocapillaris. Then, we manually moved the slab to detect the neovascular lesions, referring to the B-mode scan [23]. Two independent investigators (KA and RO) evaluated the images for the presence of CNV.

### Identification of CVH

**Fig 1:**
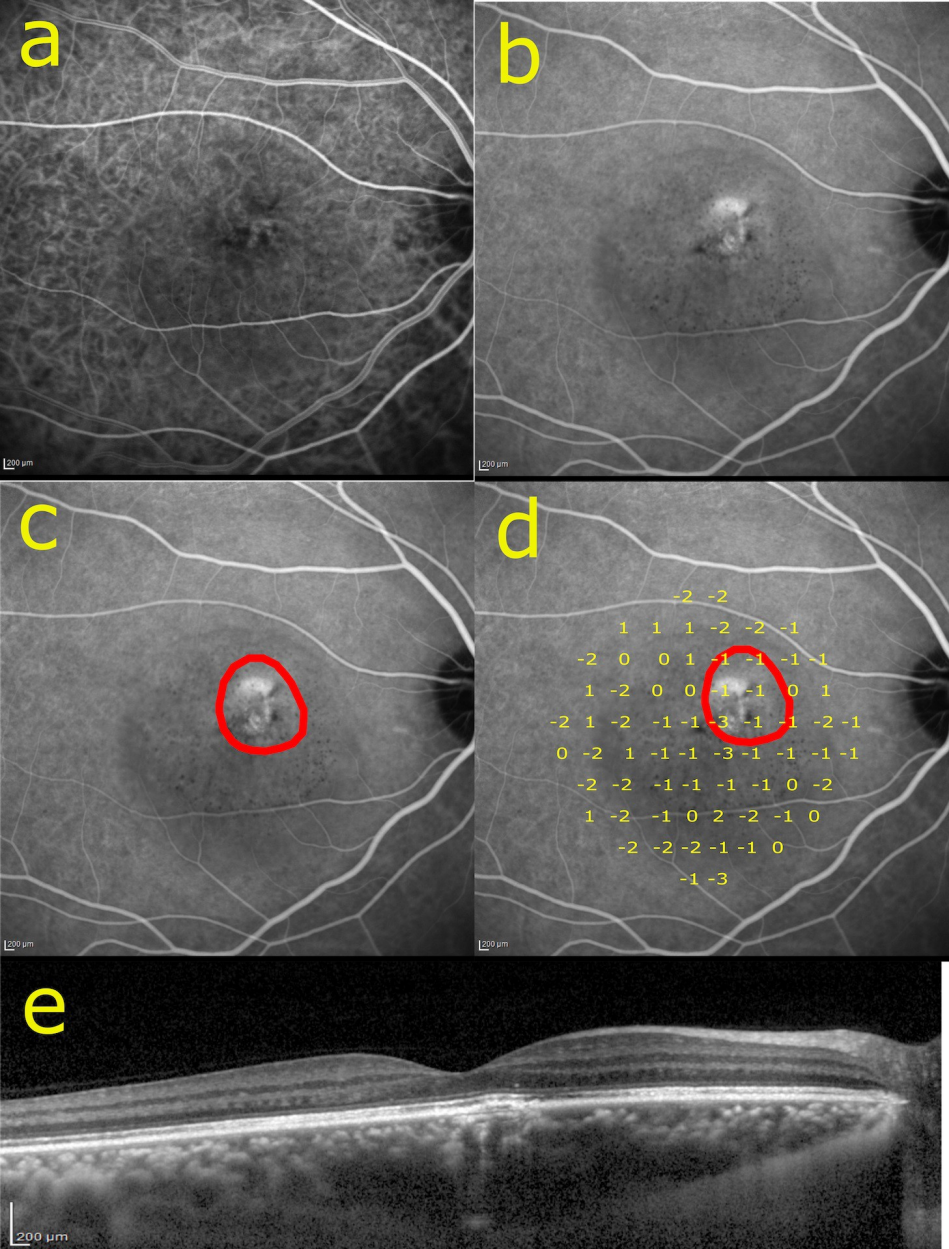
Representative images of identifying CVH by ICGA and superimposing retinal sensitivity. The right eye of a 38-year-old man that had not been accompanied with exudative changes for more than 6 months. (**a**) Early phase of ICGA in an examined eye. (**b**) Late phase of ICGA. CVH was observed nasal superior to the fovea. (**c**) Identification of CVH (red line). (**d**) Retinal sensitivity was superimposed on **c**. (**e**) EDI-OCT image of the horizontal line scan through the fovea.

CVH was evaluated using ICGA images (**Fig 1A and 1B**). On ICGA, CVH was defined as irregular areas of increased fluorescence during the mid and late phases, often surrounding dilated pachyvessels [24]. Identification of the CVH area was conducted by two experienced retinal specialists (KA and YN) (**Fig 1C**) [24,25]. The mid-phase image obtained between 5 and 6 minutes after dye injection was used for identification of CVH. We identified CVH only within the radius of 10 degrees centered on the fovea where the retinal sensitivity was measured. The measurement of the area of CVH were made by two masked graders (KA and YN) independently. To verify the inter-grader repeatability, intraclass correlation coefficient (ICC) of the values was calculated.

### 10–2 VF measurement

Each patient underwent VF testing with the AP-7000 automatic perimeter (KOWA Company Ltd., Tokyo, Japan) using a 10° radius from the fovea. A white-on-white 10–2 measurement was conducted using the Swedish interactive threshold algorithm standard test and the standard Goldmann size III stimulus and tested at 68 points. After the completion of the sensitivity testing, the sensitivity values were superimposed on the corresponding site in the ICGA image using the built-in software **(Fig 1D)**. The difference in average sensitivities between the inside and outside of CVH was calculated in each eye (⊿Retinal Sensitivity o-i; ⊿RSo-i).

### Statistical analysis

Demographic characteristics (age, sex, CCT, BCVA, the maximum height and duration of SRD) were compared between subjects with and without CNV using the chi-square test for categorical variables and the paired *t*-test for continuous variables. Mean RS within or outside CVH was calculated in the total, CVH+, or CVH− eyes. Within the eyes with CNV or those without CNV, ⊿RSo-i was analyzed using the Mann–Whitney U test. The mean ⊿RSo-i values were also compared between both groups using the Mann–Whitney U test. The relationship between ⊿RSo-i and age, sex, CCT, presence or absence of CNV, the maximum height or duration of SRD was analyzed by univariate and multivariate analyses. In univariate analysis, linear regression models were used for continuous variables, whereas logistic regression models were used for binary outcomes. Multiple explanatory variables were analyzed by multiple regression analysis, followed by model selection using the second-order bias-corrected Akaike information criterion. Hence, the optimal linear model was then selected among all possible combinations of predictors (2^4^ patterns when there were four explanatory variables). The variables included in the optimal model were considered significant. P-values less than 0.05 were considered statistically significant.

All statistical analyses were conducted using the JMP version 11.0 software program (SAS Institute, Cary, NC, USA).

## Results

In total, 12 eyes of 12 patients were included. The mean age of the patients was 52.3 ± 9.4 years (mean ± standard deviation: SD, range: 47–69 years), and there were nine male patients. The characteristics of the patients are shown in **Table 1**. CNV was detected by OCTA in six eyes (50%).

**Table 1:** Demographic characteristics of the patients.

	Total	CNV+	CNV−	p value
Case (eyes)	12	6	6	
Age (mean ± SD) [range]	52.3 ± 9.4 [47–69]	50.0 ± 11.2 [47–67]	54.7 ± 7.7 [47–69]	0.42*
Male sex (patients)	9	4	5	0.51^†^
CCT (μm)	351 ± 127	387 ± 110	315 ± 142	0.35*
BCVA (logMAR)	0.02 ± 0.12	0.002 ± 0.02	0.018 ± 0.14	0.67*
The maximum height of SRD (μm)	163 ± 65	165 ± 76	161 ± 58	0.75*
The duration of SRD (months)	3.13 ± 1.28	3.0 ± 1.10	3.25 ± 1.54	0.91*

*Mann–Whitney U test;

^†^Chi-square test.

The measurement of CVH area were made by two masked graders, independently. The inter-grader ICC was 0.932 [95% confidence interval (CI) 0.785–0.98], which was considered to be excellent.

**Fig 2:**
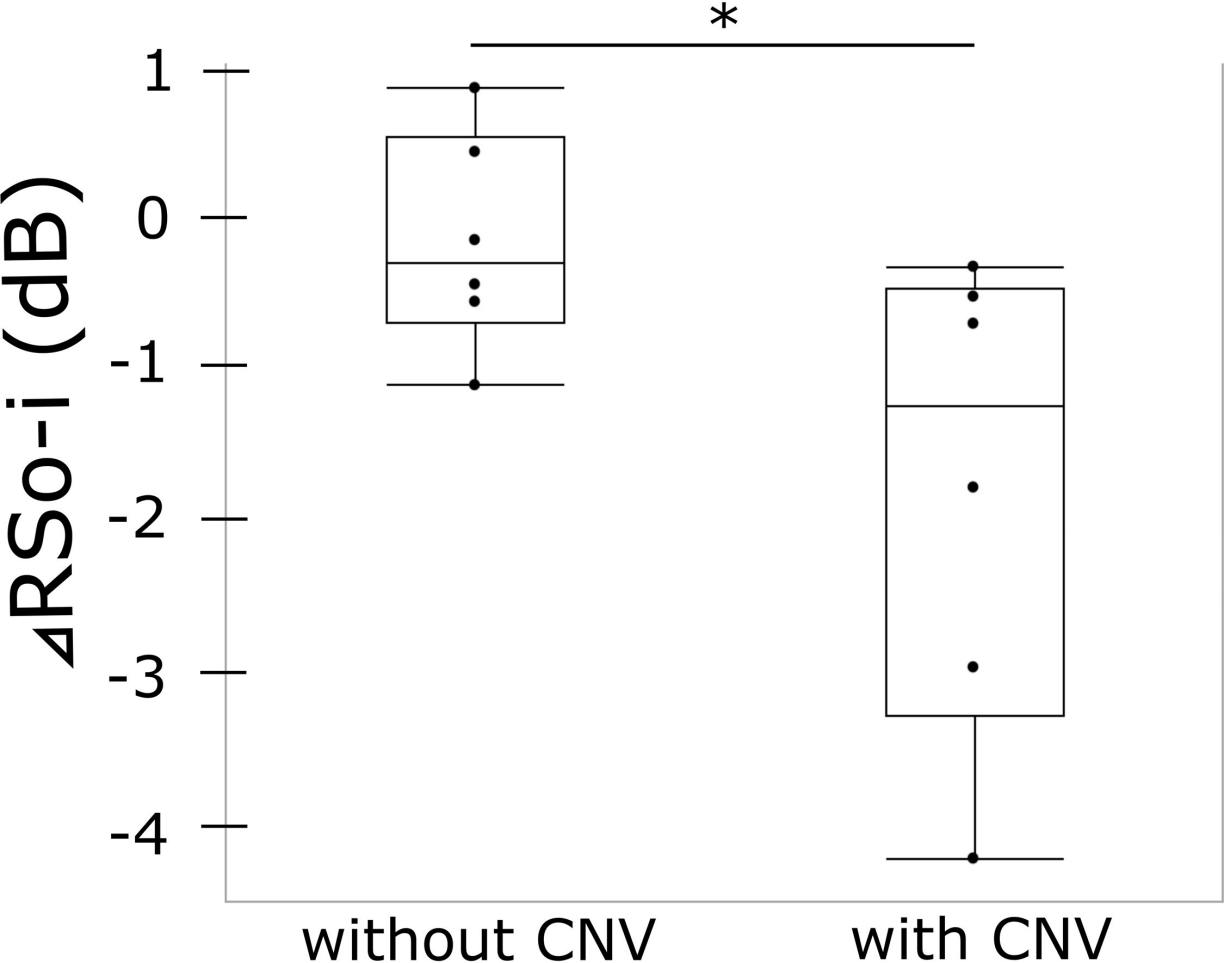
Difference in retinal sensitivity within and outside CVH (⊿RSo-i) in patients with CNV (n = 6) and without CNV (n = 6). ⊿RSo-i was significantly better in eyes without CNV than in eyes with CNV (p = 0.047).

**Fig 3:**
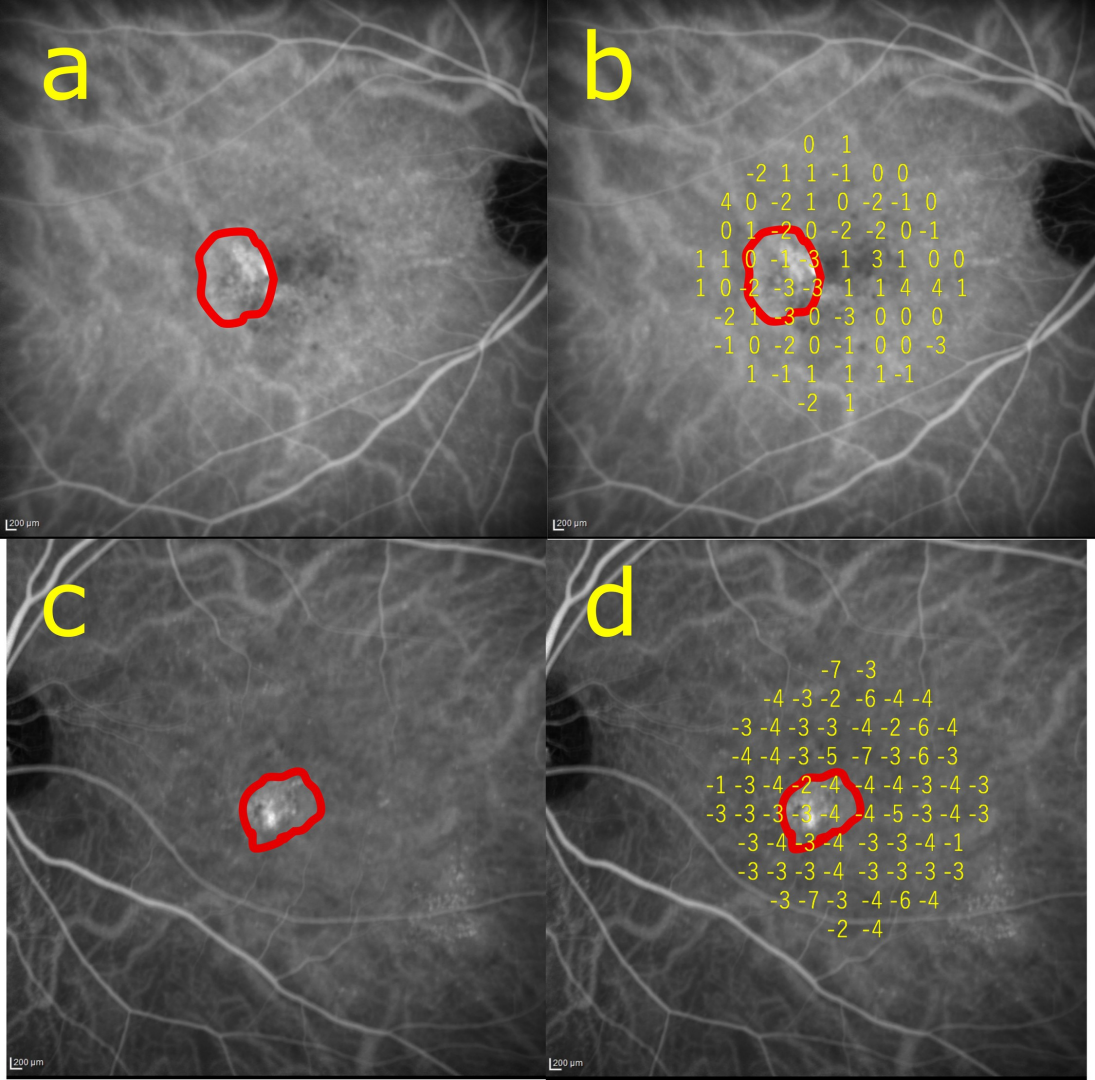
Representative images of the CVH and superimposing retinal sensitivity in eyes with or without CNV. (a,b) The right eye of a 56-year-old patient. CNV was detected within the CVH area. (a) Intermediate phase of ICGA revealed the CVH area (red line). (b) Retinal sensitivity was superimposed on a. The difference in average sensitivities between the inside and outside of CVH was -2.21. (c,d) The left eye of a 68-year-old patient. CNV was not detected within the CVH area. (c) Intermediate phase of ICGA revealed the CVH area (red line). (d) Retinal sensitivity was superimposed on c. The difference in average sensitivities between the inside and outside of CVH was -0.47.

Variables were compared between eyes with and without CNV. There was no statistical difference in age, sex, CCT, the maximum height or the duration of SRD. The RS outside CVH was similar between CNV+ and CNV− eyes, whereas the RS inside CVH was numerically lower in CNV+ eyes than in CNV− eyes. RS inside CVH was significantly lower than that outside CVH in the CNV+ group (−1.60 ± 1.51, p = 0.016) but not in the CNV− group (−0.04 ± 0.68, p = 0.64, **Table 2**). ⊿RSo-i was significantly better in eyes without CNV than in eyes with CNV eyes (p = 0.047, **Fig 2**). Representative figures were shown in **Fig 3**.

**Table 2:** The difference in average sensitivities between the inside and outside of CVH (⊿RSo-i) in eyes with CNV (CNV+, n = 6) or without CNV (CNV−, n = 6).

	Total	CNV+	CNV−	p value*
RS outside CVH	−1.44 ± 1.12	−1.37 ± 0.99	−1.50 ± 1.34	0.17
RS inside CVH	−2.25 ± 1.82	−2.95 ± 2.07	−1.54 ± 1.34	0.016
⊿RSo-i (dB) P value^‡^	−0.81 ± 1.38	−1.60 ± 1.51 0.016	−0.04 ± 0.68 0.64	0.047

*Mann–Whitney U test between groups.

^‡^Mann–Whitney U test within the eyes of each group. Statistical difference from zero was analyzed.

Additionally, above the CVH area in cases without CNV (n = 6), disruption of IZ and EZ was seen in 2 and 2 eyes, respectively. In contrast, above the CVH area in cases with CNV (n = 6), disruption of IZ and EZ was seen in 6 and 2 eyes, respectively. Of note, disruption of IZ was detected in all 6 eyes with CNV, while it was in 2 eyes without CNV.

### Association between ⊿RSo-i and demographic or anatomic factors

In multivariate analysis, the presence of CNV was included in the optimal model for ⊿RSo-i. Specifically, eyes with CNV were associated with the decrease in RS (**Table 3**).

**Table 3:** Results of the optical model selection for ⊿RSo-i.

	Univariate Analysis		Multivariate Analysis		
Parameters	coefficient	p value	coefficient	standard error	p value
Age (years)	52.3	0.20	N.S.		
Sex	3.01	0.22	N.S.		
CCT (μm)	351.2	0.48	N.S.		
CNV absent/present	0.5	0.046	-1.80	0.57	0.012
The amount SRD (μm)	163	0.57	N.S.		
The duration of SRD (months)	3.13	0.62	N.S.		

## Discussion

This study was conducted to analyze the association between CVH and RS in eyes with pachychoroid diseases, focusing on the presence of CNV. Results showed that the presence of CNV was associated with the decrease in RS.

We observed that the RS within and outside CVH (⊿RSo-i) was significantly different only in eyes with CNV. Furthermore, univariate and multivariate analyses revealed that the presence of CNV was an independent factor associated with ⊿RSo-i. Studies have reported that in pachychoroid diseases, the area of CVH is anatomically linked with atrophy or CNV [12,13]. However, there is no clear understanding of the changes in retinal function in CVH and the association with the presence or absence of CNV. Sulzbacher et al. divided chronic CSC into neovascular CSC and nonneovascular CSC [21]. They found that CNV was detected in 34.5% of eyes with chronic CSC by OCTA. Their study showed that eyes with CSC demonstrated worse distance visual acuity at the initial or final visit and worse reading acuity at the final visit in the presence of CNV [21]. These results indicate that retinal function could be worsened, especially with CNV. However, considering that the lesion does not necessarily involve the fovea, it is essential to not only compare visual acuity but also analyze the local function within or outside the lesion to examine functional changes. Therefore, we evaluated the RS within or outside the lesion and found that it was significantly decreased inside CVH but only in eyes with nonexudative CNV. The mechanism underlying the decrease in retinal function in CVH in eyes with CNV remains unclear. Considering that CNV generally develops within the CVH area, there are two hypotheses: one is that persisting detrimental process in the RPE or the sensory retina induced by CNV, although it appears quiescent, causes progressive retinal atrophy. The other hypothesis is that the CVH that could cause CNV should be more ischemic or should have more aberrant circular condition, which could result in atrophic changes in the retina and the RPE. However, our study could not address this point because of its retrospective design. A further prospective study may elucidate a more detailed course of functional changes or the cause-and-effect relationship of the functional changes in the retina and the development of CNV. Furthermore, it is of much interest to investigate distinct retinal sensitivity within or without CNV area. However, the visual field sensitivity test used in this study covered 10 degrees in radius with 68 points, and the distance between the measurement points was approximately 2 degrees (almost equivalent of 600 micrometers). Considering the size or distorted shape of the CNV, it was impossible to distinguish the sensitivity inside and outside the CNV accurately. Therefore, it should be helpful to investigate the RS with more densely distributed measurement points for understanding the structure–function relationships between CNV and retinal sensitivity in more detail.

In eyes without CNV, RS were comparable in terms of between the inside and outside of CVH. To the best of our knowledge, there have been no reports investigating the RS changes in CVH. The presence of CVH suggests vascular congestion and choriocapillaris damage, leading to relative choroidal ischemia [1]. The changes are more common in eyes with CSC and PPE than in eyes with uncomplicated pachychoroid diseases [26]. Hence, CVH could indicate a pathological condition that predisposes to CSC or PPE. However, the results of the present study suggest that CVH per se may not lead to retinal dysfunction.

To elucidate the relationship between the presence of CNV and retinal sensitivity decrease, we assessed the integrity of EZ or IZ above the CVH. Consequently, the eyes with CNV tended to be more likely to show disrupted IZ than the eyes without CNV. The results might support the hypothesis that the eyes with CNV could be more likely to cause the impairment of the photoreceptor within CVH area than the eyes without CNV, leading to relative decrease in retinal sensitivity.

There were several limitations in our study. First, the small sample size was a major limitation. Our sample size might be responsible for a lack of power to reveal verification of associations between factors that were not found to be statistically significant in the current cohort. Additionally, although there was statistically significant association between the presence of CNV and the reduction of retinal sensitivity in the univariate and the multivariate analysis in the present study, a future analysis with larger samples should be necessary to confirm the results in the current analysis. Second, it was a retrospective study, which may have led to selection bias. Furthermore, influence of the previous exudative changes over six months before examination should be considered as a bias. Thus it would be ideal to investigate eyes with or without quiescent CNV that were associated with pachychoroid but have never experienced SRD. Because of the retrospective design of the current study, however, it was very difficult to recruit such cases because it is impossible to assure that they never experienced SRD before the first visit at our clinic. Therefore, we left that out of the focus in the current study. However, because these changes generally evolve irrespective of the CVH lesion, the RS changes within CVH could not be explained completely by these exudative changes but, at least in part, by the atrophic changes in CVH per se. We believe that the presence or absence of nonexudative CNV in CVH would provide more information. Additionally, in order to explain the mechanism underlying the decrease in retinal sensitivity in CVH in eyes with CNV, it would be helpful to compare the retinal sensitivity and the microstructure of the retina or the choroid in point-to-point manner. For this purpose, more detailed measurement of microperimeter and OCT would be required.

## Conclusions

The RS within CVH was decreased in patients with nonexudative CNV. Detecting quiescent CNV by OCTA may be helpful in suspecting retinal dysfunction in CVH in patients with pachychoroid diseases.

## Supporting information

S1 File (XLSX)

## References

[1] Cheung CMG, Lee WK, Koizumi H, Dansingani K, Lai TYY, Freund KB. Pachychoroid disease. Eye (Lond). 2019;33: 14–33. doi: 10.1038/s41433-018-0158-4 29995841 PMC6328576

[2] Yanagi Y. Pachychoroid disease: a new perspective on exudative maculopathy. Jpn J Ophthalmol. 2020;64: 323–337. doi: 10.1007/s10384-020-00740-5 32318919

[3] Yamashiro K, Hosoda Y, Miyake M, Ooto S, Tsujikawa A. Characteristics of Pachychoroid Diseases and Age-Related Macular Degeneration: Multimodal Imaging and Genetic Backgrounds. J Clin Med. 2020;9. doi: 10.3390/jcm9072034 32610483 PMC7409179

[4] Margolis R, Spaide RF. A pilot study of enhanced depth imaging optical coherence tomography of the choroid in normal eyes. Am J Ophthalmol. 2009;147: 811–815. doi: 10.1016/j.ajo.2008.12.008 19232559

[5] Warrow DJ, Hoang QV, Freund KB. Pachychoroid pigment epitheliopathy. Retina. 2013;33: 1659–1672. doi: 10.1097/IAE.0b013e3182953df4 23751942

[6] Fung AT, Yannuzzi LA, Freund KB. Type 1 (sub-retinal pigment epithelial) neovascularization in central serous chorioretinopathy masquerading as neovascular age-related macular degeneration. Retina. 2012;32: 1829–1837. doi: 10.1097/IAE.0b013e3182680a66 22850219

[7] Pang CE, Freund KB. Pachychoroid neovasculopathy. Retina. 2015;35: 1–9. doi: 10.1097/IAE.0000000000000331 25158945

[8] Takahashi A, Ooto S, Yamashiro K, Tamura H, Oishi A, Miyata M, et al. Pachychoroid Geographic Atrophy: Clinical and Genetic Characteristics. Ophthalmol Retina. 2018;2: 295–305. doi: 10.1016/j.oret.2017.08.016 31047238

[9] Imamura Y, Fujiwara T, Margolis R, Spaide RF. Enhanced depth imaging optical coherence tomography of the choroid in central serous chorioretinopathy. Retina. 2009;29: 1469–1473. doi: 10.1097/IAE.0b013e3181be0a83 19898183

[10] Lee WK, Baek J, Dansingani KK, Lee JH, Freund KB. CHOROIDAL MORPHOLOGY IN EYES WITH POLYPOIDAL CHOROIDAL VASCULOPATHY AND NORMAL OR SUBNORMAL SUBFOVEAL CHOROIDAL THICKNESS. Retina. 2016;36 Suppl 1: S73–S82. doi: 10.1097/IAE.0000000000001346 28005665

[11] Pang CE, Freund KB. Pachychoroid pigment epitheliopathy may masquerade as acute retinal pigment epitheliitis. Invest Ophthalmol Vis Sci. 2014;55: 5252. doi: 10.1167/iovs.14-14959 25149971

[12] Miyake M, Tsujikawa A, Yamashiro K, Ooto S, Oishi A, Tamura H, et al. Choroidal neovascularization in eyes with choroidal vascular hyperpermeability. Invest Ophthalmol Vis Sci. 2014;55: 3223–3230. doi: 10.1167/iovs.14-14059 24781946

[13] Kim JH, Chang YS, Lee TG, Kim CG. Choroidal vascular hyperpermeability and punctate hyperfluorescent spot in choroidal neovascularization. Invest Ophthalmol Vis Sci. 2015;56: 1909–1915. doi: 10.1167/iovs.14-16000 25722216

[14] Miyake M, Ooto S, Yamashiro K, Takahashi A, Yoshikawa M, Akagi-Kurashige Y, et al. Pachychoroid neovasculopathy and age-related macular degeneration. Sci Rep. 2015;5: 16204. doi: 10.1038/srep16204 26542071 PMC4635432

[15] Laiginhas R, Yang J, Rosenfeld PJ, Falcão M. Nonexudative Macular Neovascularization—A Systematic Review of Prevalence, Natural History, and Recent Insights from OCT Angiography. Ophthalmol Retina. 2020;4: 651–661. doi: 10.1016/j.oret.2020.02.016 32335033 PMC7354220

[16] Chen L, Messinger JD, Sloan KR, Swain TA, Sugiura Y, Yannuzzi LA, et al. Nonexudative Macular Neovascularization Supporting Outer Retina in Age-Related Macular Degeneration: A Clinicopathologic Correlation. Ophthalmology. 2020;127: 931–947. doi: 10.1016/j.ophtha.2020.01.040 32247535

[17] Yanagi Y, Mohla A, Lee W-K, Lee SY, Mathur R, Chan CM, et al. Prevalence and Risk Factors for Nonexudative Neovascularization in Fellow Eyes of Patients With Unilateral Age-Related Macular Degeneration and Polypoidal Choroidal Vasculopathy. Invest Ophthalmol Vis Sci. 2017;58: 3488–3495. doi: 10.1167/iovs.16-21167 28702676

[18] Carnevali A, Capuano V, Sacconi R, Querques L, Marchese A, Rabiolo A, et al. OCT Angiography of Treatment-Naïve Quiescent Choroidal Neovascularization in Pachychoroid Neovasculopathy. Ophthalmol Retina. 2017;1: 328–332. doi: 10.1016/j.oret.2017.01.003 31047519

[19] Savastano MC, Rispoli M, Lumbroso B. THE INCIDENCE OF NEOVASCULARIZATION IN CENTRAL SEROUS CHORIORETINOPATHY BY OPTICAL COHERENCE TOMOGRAPHY ANGIOGRAPHY. Retina. 2021;41: 302–308. doi: 10.1097/IAE.0000000000002810 32310626 PMC7819522

[20] Yanagi Y, Mohla A, Lee SY, Mathur R, Chan CM, Yeo I, et al. Incidence of Fellow Eye Involvement in Patients With Unilateral Exudative Age-Related Macular Degeneration. JAMA Ophthalmol. 2018;136: 905–911. doi: 10.1001/jamaophthalmol.2018.2154 29879284 PMC6142947

[21] Sulzbacher F, Schütze C, Burgmüller M, Vécsei-Marlovits PV, Weingessel B. Clinical evaluation of neovascular and non-neovascular chronic central serous chorioretinopathy (CSC) diagnosed by swept source optical coherence tomography angiography (SS OCTA). Graefes Arch Clin Exp Ophthalmol. 2019;257: 1581–1590. doi: 10.1007/s00417-019-04297-z 31037488

[22] Hood DC, Ramachandran R, Holopigian K, Lazow M, Birch DG, Greenstein VC. Method for deriving visual field boundaries from OCT scans of patients with retinitis pigmentosa. Biomed Opt Express. 2011;2: 1106–1114. doi: 10.1364/BOE.2.001106 21559123 PMC3087568

[23] Asano-Shimizu K, Asano S, Murata H, Azuma K, Nomura Y, Inoue T, et al. Early changes of vascular lesions and responses to combined photodynamic therapy in patients with polypoidal choroidal vasculopathy. Int Ophthalmol. 2020;40: 1335–1345. doi: 10.1007/s10792-020-01299-3 32026179

[24] Yanagi Y, Ting DSW, Ng WY, Lee SY, Mathur R, Chan CM, et al. CHOROIDAL VASCULAR HYPERPERMEABILITY AS A PREDICTOR OF TREATMENT RESPONSE FOR POLYPOIDAL CHOROIDAL VASCULOPATHY. Retina. 2018;38: 1509–1517. doi: 10.1097/IAE.0000000000001758 28704255

[25] Sakurada Y, Leong BCS, Parikh R, Fragiotta S, Freund KB. ASSOCIATION BETWEEN CHOROIDAL CAVERNS AND CHOROIDAL VASCULAR HYPERPERMEABILITY IN EYES WITH PACHYCHOROID DISEASES. Retina. 2018;38: 1977–1983. doi: 10.1097/IAE.0000000000002294 30198969

[26] Ersoz MG, Arf S, Hocaoglu M, Sayman Muslubas I, Karacorlu M. INDOCYANINE GREEN ANGIOGRAPHY OF PACHYCHOROID PIGMENT EPITHELIOPATHY. Retina. 2018;38: 1668–1674. doi: 10.1097/IAE.0000000000001773 28723851

